# The Medicinal Properties of Woodall Spa Waters

**Published:** 1887-02-12

**Authors:** 


### THE MEDICINAL PROPERTIES OF WOODHALL SPA WATERS.

Enquirer writes from North Wales:—"Will you allow me to ask in your Journal, if anyone can give reliable information as to the curative and medicinal properties of the mineral waters at Woodhall Spa, Lincolnshire, which is spoken of as likely to become the 'great health-resort of the future'? I am particularly anxious to know whether the waters are likely to be beneficial in glandular disease."

*** Can anyone reply fully to this query?—Ed. T. H.

Great Northern Central Hospital, N.—The number of patients relieved during the month of January was:—Medical, 1,003; Surgical, 1,346; total, 2,349; of which 746 were new cases; 173 accidents; and 28 were not able to be admitted for want of room.

Hull Royal Infirmary.—The Working Men's Committee connected with the above Institution have contributed the following sums towards the expenses during the year 1886:—

| | £ | s. | d. |
|---|---|---|---|
| Donations from Workshops - - - | 976 | 16 | 1 |
| Proceeds of a Concert - - - - | 36 | 10 | 9 |
| Hospital Saturday Committee - - | 299 | 9 | 3 |
| | 1,312 | 16 | 1 |

